# COVITALE 2020 from eastern Indian population: imageologists perspective, a learning curve

**DOI:** 10.1186/s43055-021-00634-7

**Published:** 2021-10-06

**Authors:** Kamal Kumar Sen, Roopak Dubey, Mayank Goyal, Humsheer Sethi, Ajay Sharawat, Rohit Arora

**Affiliations:** Department of Radiodiagnosis, Kalinga Institute of Medical Sciences, KIIT Road, Patia, Bhubaneswar, Odisha 751024 India

**Keywords:** COVID-19, Artificial intelligence, Lung ultrasonography, X-rays

## Abstract

**Background:**

High-resolution computed tomography (HRCT) chest becomes a valuable diagnostic tool for identifying patients infected with Coronavirus Disease 2019 (COVID-19) in the early stage, where patients may be asymptomatic or with non-specific pulmonary symptoms. An early diagnosis of COVID-19 is of utmost importance, so that patients can be isolated and treated in time, eventually preventing spread of the disease, improving the prognosis and reducing the mortality. In this paper, we have highlighted our radiological experience of dealing with the pandemic crisis of 2020 through the study of HRCT thorax, lung ultrasonography, chest X-rays and artificial intelligence (AI).

**Results:**

Results of CT thorax analysis have been given in detail. We had also compared CT severity score (CTSS) with clinical and laboratory parameters. Correlation of CTSS with SpO2 values and comorbidities was also studied. We also analysed manual CTSS with the CTSS scored calculated by the AI software.

**Conclusions:**

CTSS and use of COVID-19 Reporting and Data System (CORADS) result in accuracy and uniform percolation of information among the clinicians. Bed-side X-rays and ultrasonography have played a role where the patients could not be shifted for CT scan. The possibility of predicting impending or progression of hypoxia was not possible when SpO2 mapping was correlated with the CTSS. AI was alternatively tried with available software (*CT pneumonia analysis*) which was not so appropriate considering the imaging patterns in the bulk of *atypical category*.

## Background

A febrile lower respiratory infection of unknown aetiology was reported in Wuhan, China, in December 2019 and was later discovered to be caused by a novel coronavirus, a severe acute respiratory syndrome coronavirus 2 (SARS-CoV-2) [1]. Since then, it has spread fast over the world, infecting millions of people [2].

Because it has already been shown that high-resolution computed tomography (HRCT) chest has excellent accuracy for early diagnosis, triage, proper patient management and follow-up, radiology has become a pioneer branch for identifying individuals infected with Coronavirus Disease 2019 (COVID-19) [3, 4]. Artificial intelligence (AI)-assisted imaging acquisition may substantially assist in automating the scanning approach and reshaping the workflow with little patient interaction, ensuring the greatest protection for imaging specialists. In addition, AI can boost productivity by accurately delineating infections in X-ray and CT images, allowing for more efficient quantification [5].

Since most lung parenchymal lesions in COVID-19 are distributed peripherally, these lesions may be visualized on lung ultrasonography (LUS) in most of the cases [6, 7].

The main aim of this article is to share overall radiological experience in management of COVID-19. We looked at the under-appreciated modality, lung ultrasonography and the overhyped artificial intelligence in COVID patients, in addition to HRCT of thorax and X-rays.

## Methods

### Study design

This prospective observational study was done at Odisha COVID Hospital, KIMS, conducted over a period of 10 months between March and December 2020. Ethical committee clearance has been taken for the research purpose. A study of 4338 COVID-19 patients who had undergone HRCT chest was scrutinized by experienced radiologists, blindfolded to any other associated findings. Out of these 4338 patients, 2000 images were also scrutinized with AI software. Seriously ill-intensive care unit (ICU) patients (124 patients) were studied by lung ultrasound and X-rays helped in 1402 patients.

The conventional gold standard was real-time reverse transcription polymerase chain reaction (rRT-PCR) test, for the qualitative detection of nucleic acid from SARS-CoV-2 in upper and lower respiratory specimens (such as nasopharyngeal or oropharyngeal swabs, sputum, lower respiratory tract aspirates or bronchoalveolar lavage).

### Inclusion criteria

For HRCT thorax, the inclusion criterion was laboratory-proven RT-PCR-positive cases. Only critically sick ICU patients who were unable to undergo HRCT were considered for LUS.

### Exclusion criteria

Clinically suspected COVID-19 cases that were negative on RT-PCR, as well as COVID-19-positive patients who had not underwent HRCT chest, were eliminated from the research. Pregnant females and patients with severe artefacts on CT images were also excluded.

### Epidemiological and clinical data collection

All relevant epidemiological data were collected from hospital records. Based on the clinical data, the patients were divided into asymptomatic and symptomatic groups. The patients were then categorized into three groups (mild, moderate and severe), based on the clinical severity (Table 1).

**Table 1:** Clinical severity score according to Ministry of Health and Family Welfare, Government of India

Clinical severity	Symptoms/signs	Clinical parameters
Mild	Mild symptoms of URTI	O2 saturation: (95–100%)
Moderate	Pneumonia RR > 24/min	O2 saturation: (90–94%)
Severe	Pneumonia/ARDS/sepsis/shock RR > 30/min	O2 saturation: (< 90%)

### CT image data acquisition

Examination of the patients with HRCT chest was performed with dedicated 64 Slice CT scanner (Siemens, Somatom: Go up). Scanning parameters were 1 mm slice thickness, 5 mm gap, 120 kV and 150 mA. The scan was done generally within 2 days of hospitalization.

### Image interpretation and analysis

The HRCT chest images were viewed with standard lung (window width 1500 HU; window level – 600 HU) and mediastinal (window width 350 HU; window level 40 HU) settings.

Findings on HRCT chest were classified into four patterns—*Typical, Indeterminate, Atypical and Negative* for COVID-19 pneumonia (Table 2) [8].

**Table 2:** Reporting language by RSNA for CT findings related to COVID-19

Classification	CT findings	Rationale
Typical appearance	Peripheral, bilateral, GGO with or without consolidation or visible intralobular lines (“crazy-paving”) Multifocal GGO of rounded morphology with or without consolidation or visible intralobular lines (“crazy-paving”) Reverse halo sign or other findings of organizing pneumonia (seen later in the disease)	Commonly reported imaging features of greater specificity for COVID-19 pneumonia
Indeterminate appearance	Absence of typical features AND presence of: Multifocal, diffuse, perihilar or unilateral GGO with or without consolidation lacking a specific distribution and are non-rounded or non-peripheral Few very small GGO with a non-rounded and non-peripheral distribution	Non-specific imaging features of COVID-19 pneumonia
Atypical appearance	Absence of typical or indeterminate features and presence of: Isolated lobar or segmental consolidation without GGO Discrete small nodules (centrilobular,“tree-in bud”) Lung cavitation Smooth interlobular septal thickening with pleural effusion	Uncommonly or not reported features of COVID-19 pneumonia
Negative for pneumonia	No CT features to suggest pneumonia	No features of pneumonia

Semi-quantitative assessment for extent of lung parenchymal involvement in CT chest images was done using CT severity score (CTSS). Each lobe of the lungs (total five lobes) was visually assessed and given a score between 0 and 5 on the basis of percentage of parenchymal involvement: 1 for < 5% involvement, 2 for 5–25% involvement, 3 for 26–49% involvement, 4 for 50–75% involvement and 5 for > 75% involvements. The CTSS is the sum of all lobar scores and can range from 0 (no involvement) to 25 (maximum involvement).

Changes in CT findings with duration of symptoms were assessed by classifying the study population into three phases (early, intermediate and late phase), based on the duration between day of onset of illness (day of onset of symptoms in symptomatic patients or day of becoming positive on RT-PCR in asymptomatic patients) and day of CT scan. Patients were considered to be in the (a) early phase of illness if the duration was < 5 days, (b) intermediate phase of illness for 6–10 days duration and (c) late phase if the scan was done > 10 days after the date of onset of illness.

“Choicemmed MD300C2 Pulse Oximeter” was used to measure patient’s SpO2 level at the time of HRCT thorax and again after 5 days.

### Automated processing of chest CT

Artificial intelligence-based algorithm *“CT pneumonia analysis”* based on 3D deep learning developed by Siemens Healthineers was used in our study. This algorithm identifies and quantifies the hyperdense areas (areas of high opacity) of lungs. It performs automated lung opacity analysis on axial CT data with slice thicknesses up to 5 mm, resulting in multiplanar reformatting (MPR) series containing segmentations of the high opacity abnormalities of the lungs and provides a table with various measurements, e.g. the relative (“percentage of opacities”) and absolute volume of the lungs affected by opacities. Additionally, the mean and standard deviation of Hounsfield unit (HU) values between lung parenchyma and the detected opacities can be compared. Segmentation was done using multi-scale deep reinforcement learning and deep image-to-image network (DI2IN) [9, 10]. The algorithm quantifies the commonly seen CT abnormalities (i.e. GGO and consolidation) and categorized the data into two combined severity measures: (a) lung severity score (LSS), lung high opacity score (LHOS) and (b) percentage of opacity (PO), percentage of high opacity (PHO) [11]. Lung severity score, which measures the extent of lung involvement, is the sum of the individual lobe severity scores. All voxels within the lung containing either full or partial GGO or consolidations are considered as positive and given a single label and rest of images within and outside of lungs are defined as negative.

The Pearson’s correlation coefficient (*r*) was used to demonstrate linear relationship between predicted and manual (actual) values. The degree of correlation was assessed using interclass correlation (ICC) coefficient based on single rater, consistency and two-way random effect model. In order to further assess the level of agreement between two methods of LSS, Bland–Altman analysis was used. ANOVA test was used to find the difference in the categorical data between the three clinical severity groups and a two-tailed *p* value was calculated. *p* Value between the categorical data less than 0.05 indicated statistical significant difference.

### Lung ultrasonography (LUS) examination

Lung ultrasound was performed with a convex (2–6-MHz) transducer set at depth of 6.8 cm and linear transducer (10–15-MHz) set at depth of 11.6 cm. The LUS examination was performed by two radiology residents both having experience of 2.5 years. The intraclass correlation coefficient (ICC) was determined. An almost perfect agreement for the Lung segmental scores (LUSS) was noted (ICC 0.82, 95% CI 0.57–0.95).

The thorax was scanned in 12 lung areas [12–14]. The COVID-19 LUS in emergency protocol (CLUE) [15] was used. The LUSS points ranged from (normal) 0 to 3, with higher points allocated to severe lung changes. Based on the total score from 12 lung zones, the severity was classified as mild (score 1–5), moderate (> 5–15) and severe (> 15). Follow-up LUS examinations were performed in all patients after 7 (mean) days of the initial LUS examination. The LUS images were electronically stored and analysed.

The ICC was used to assess the degree of agreement between LUSS and CT segmental scoring (CT SS). An ICC < 0.50 was considered poor that from 0.50 to 0.75 moderate, that from 0.75 to 0.90 good and that from 0.90 to 1 excellent. Mean values were reported along with 95% CIs. Statistical significance was set at *p* < 0.05 using McNemar's Chi-square test. Cohen’s kappa (k) test was used to compare abnormal chest CT findings with abnormal LUS findings using the scoring systems.

### X-ray examination

All chest X-rays (CXR) were acquired with portable X-ray units (100 mA Siemens Multimobile 2.5) and processed with Care stream DV 6950 CR system. CXR were obtained in the postero-anterior (PA) or antero-posterior (AP) projection as per the condition of patient.

## Results

**Fig. 1:**
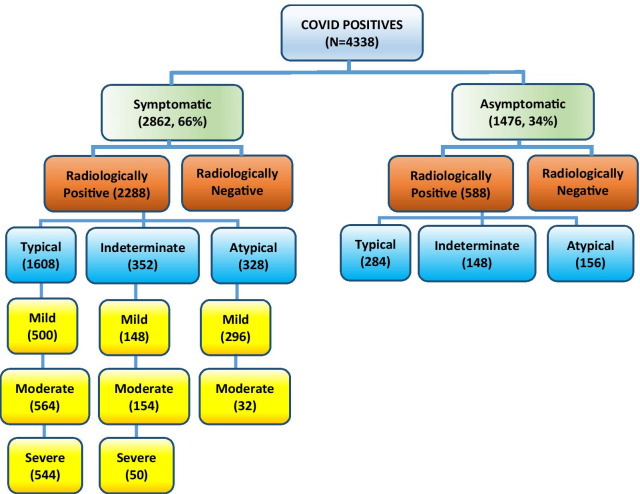
Distribution of study population according to clinical and radiological findings

**Fig. 2:**
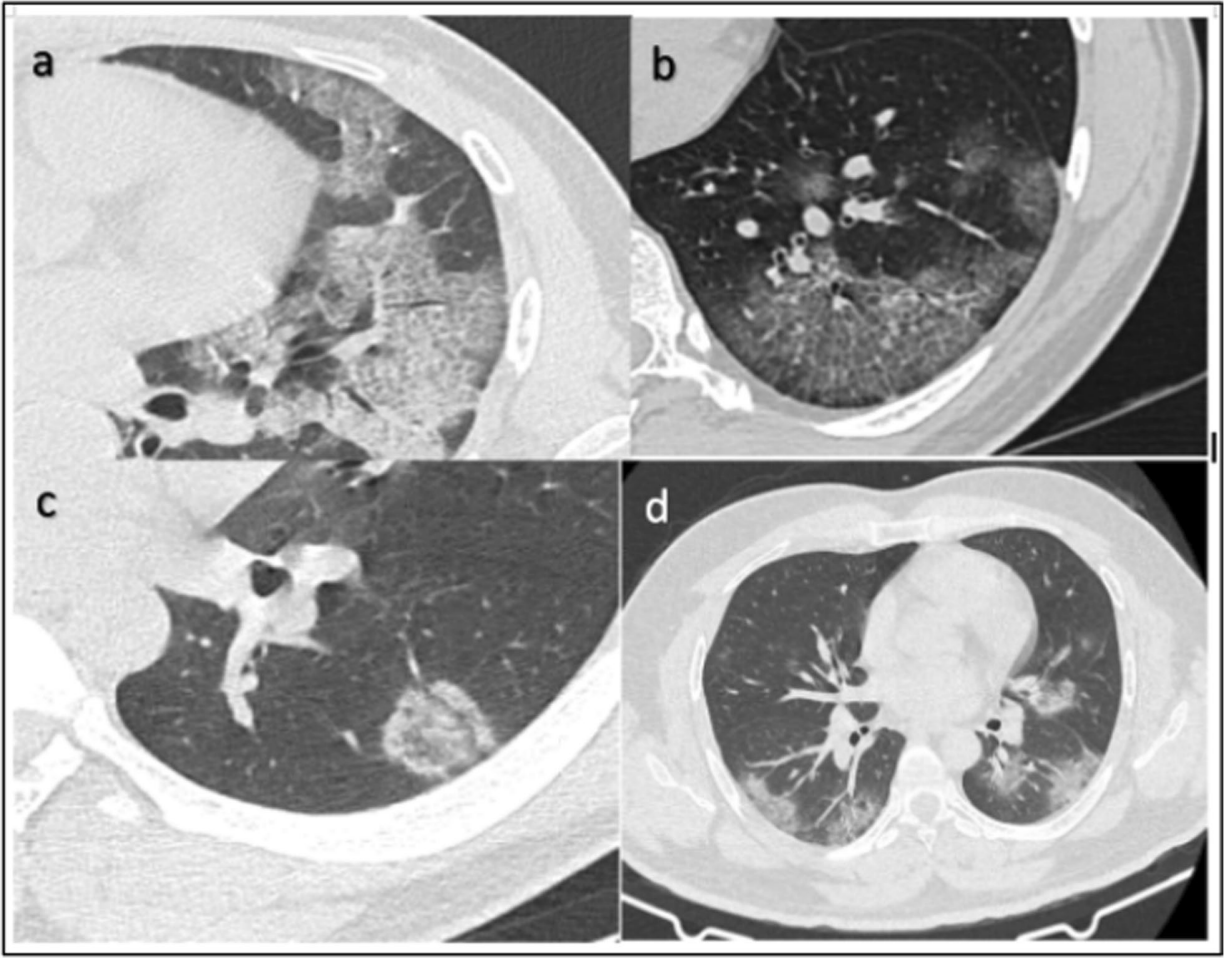
Axial HRCT images of different patients showing typical imaging features in COVID-19—crazy paving pattern (**a**, **b**), reverse halo sign (**c**) and peripherally arranged ground-glass opacities (**d**)

**Fig. 3:**
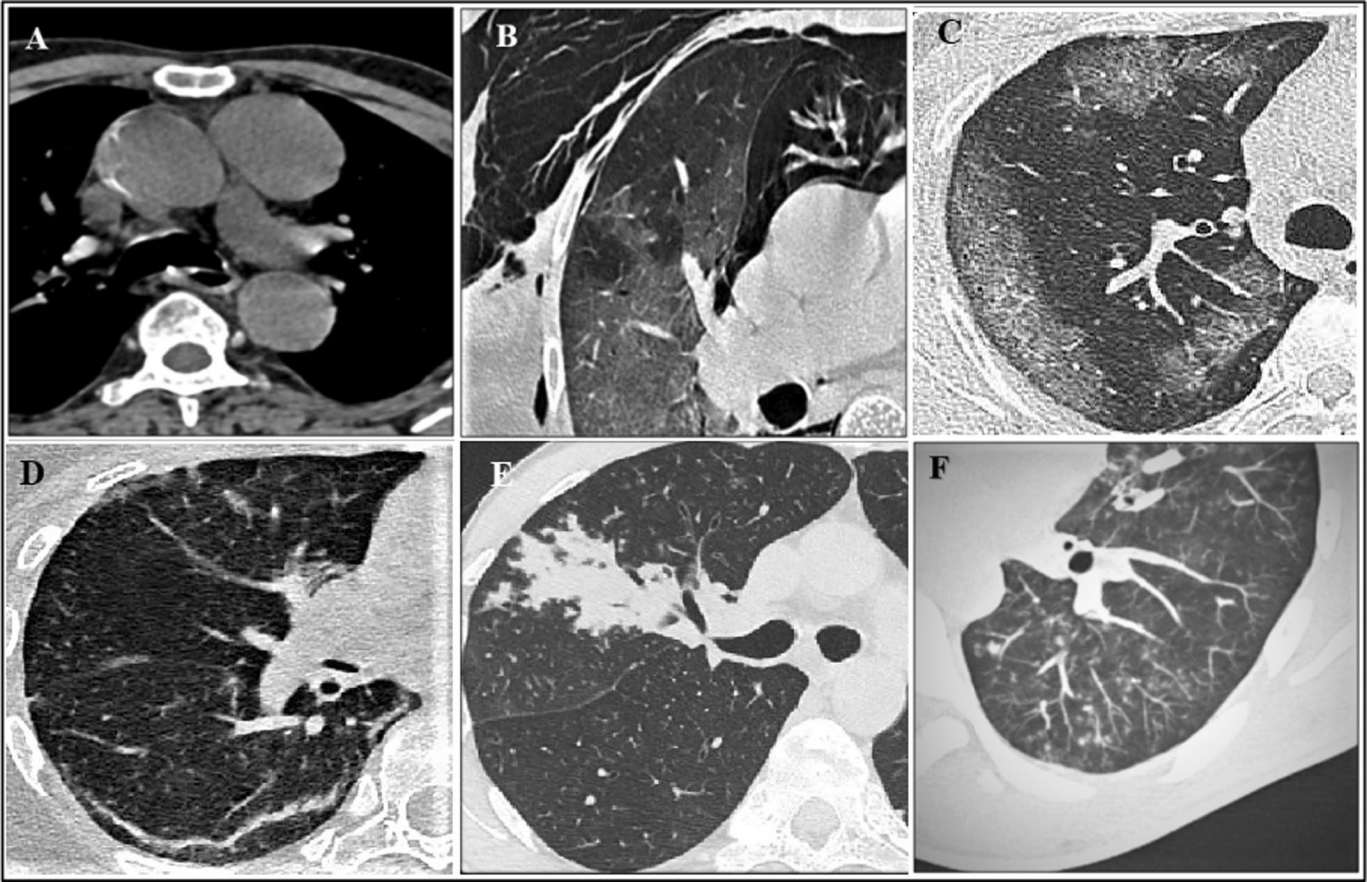
Axial section through HRCT chest of different patients demonstrating atypical imaging features. **A** Mediastinal lymphadenopathy; **B** ground-glass opacifications, pneumothorax, pneumomediastinum and subcutaneous emphysema; **C** peripheral confluent ground-glass opacifications; **D** subpleural curvilinear opacifications; **E** isolated segmental consolidation; **F** tree-in bud nodules

Serial data from 4338 COVID-19-positive patients, qualifying the inclusion criteria, were compiled and analysed (Fig. 1). In our study, the mean age of patients was 39.4 years, with majority, i.e. 2268 (52.3%), in the 21–40-year age group. Males (78.4%) outnumbered the females (21.6%). Various chest lesions, lobar involvement and distribution of lung opacities in radiologically positive patients (2876) are shown in Table 3. Various imaging features of COVID-19 pneumonia are shown in Figs. 2 and 3.

**Table 3:** Chest lesions, lobar involvement and distribution of lung opacities in radiologically positive (2876) patients

CT features	No. of patients	% among total patients(*n* = 4338)	% among radiologically positive patients (*n* = 2876
*Parenchymal opacifications*			
Only GGO present	1224	28.2	42.6
Both GGO and consolidation	1604	37	55.8
Only consolidation	48	1.1	1.6
*Associated findings*			
Subpleural bands/Atelectasis/fibrotic stripes	1208	27.8	42
Septal thickening (crazy paving and reticular pattern)	874	20.1	30.4
Vascular widening	1038	23.9	36.1
Pulmonary nodules	618	14.2	21.4
Thoracic lymphadenopathy	244	5.62	8.48
Pleural effusion	94	2.1	2
Cavitatory lesion	4	0.09	0.13
Pleural thickening	66	1.52	2.29
Bronchiectasis	54	1.24	1.64
Pneumomediastinum	4	0.091	0.13
Pulmonary emphysema	50	1.2	1
*Opacity distribution*			
Central	50	1.1	2
Peripheral	1266	29.1	53
Both	1076	24.8	45
*No. Of lobes affected*			
1 lobe	482	11.11	16.75
2 or more lobes	2394	88.89	83.25
B/L lung disease	1916	44.16	66.62
U/L lung disease	476	16.5	10.97
*Frequency of lobe involvement*			
RUL	1320	30.42	45.89
RML	1168	26.92	40.61
RLL	1750	40.34	60.8
LUL	1318	30.38	45.82
LLL	1680	38.72	58.41
AL	1272	29.32	44.22

GGO—Ground-glass opacities; B/L—bilateral; U/L—unilateral; RUL—right upper lobe; RML—right middle lobe; RLL—right lower lobe; LUL—left upper lobe; LLL—left lower lobe; AL—all lobes

All images were reviewed independently by three radiologists (5, 5 and 30 years of experience). Using intraclass correlation coefficients, comparisons between observers for HRCT thorax analysis showed acceptable interobserver reliability (ICC 0.82, 95% CI 0.74–0.89). Average CTSS in this study was found to be 4.9. Among asymptomatic patients, 86.6% cases had CTSS of zero and 13.4% had scores more than zero. 91.3% of cases having score ≤ 7 were asymptomatic or mild. On the other hand, 75.1% cases having CTSS of 8–14 were clinically moderate and 81.2% cases having score ≥ 15 were clinically severe (Table 4). So, the positive predictive values (PPVs) of CTSS ≥ 15 for detecting clinically severe and symptomatic patients are 81.2% and 98.5%, respectively. The association between CTSS grading and clinical severity was found to be statistically significant (Chi-square value = 2654, *df* = 9, *p* < 0.001).

**Table 4:** CT severity score versus clinical severity in COVID-19 patients

CT severity score (CTSS)	Clinical severity			
	Asymptomatic	Mild	Moderate	Severe
0	1278	640	22	6
1–7	156	750	174	68
8–14	34	48	544	98
15–25	8	34	56	422
Total	1476	1472	796	594

^*^Chi-square value = 2654, *df* = 9, *p* < 0.001, Significant

### Temporal changes in CT findings with duration of symptoms

**Fig. 4:**
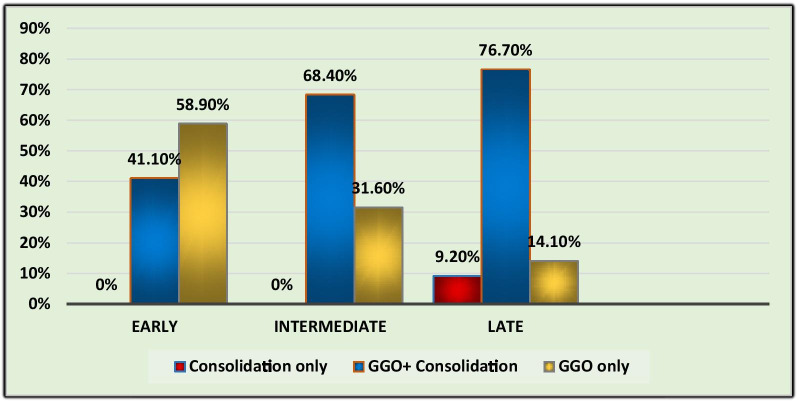
Parenchymal opacification according to phases of disease

Among the radiologically positive cases, 1492 (51.9%) patients were in early phase (≤ 7 days), 860 patients (29.9%) in intermediate phase (8–14 days) and 524 patients (18.2%) in late phase (> 14 days) of disease progression. Parenchymal opacification according to phases of disease is shown in Fig. 4. Bilateral lung involvement was seen in 39.8% of early phase patients, 81.9% of intermediate and 76.2% late phase patients. The percentage of patients in early, intermediate and late stages of disease having both peripherally and centrally distributed parenchymal lesions were 19%, 63% and 54%. The average numbers of lobes involved in early, intermediate and late phases were 2.3, 4.7 and 4.1, respectively. Average CTSS in early, intermediate and late phases among radiologically positive patients is 4.2, 14.3 and 13.1, respectively. Majority of cases with septal thickening (72.7%) are seen in patients of intermediate phase. Subpleural band-like opacities were seen in all phases of disease but are predominant in intermediate phase (40.9% cases) and late phases (79%). Pleural effusion was found in 80 cases in the intermediate phase and 14 cases in late phase of disease. Bronchiectatic changes were seen predominantly in patients of late phase (7% cases).

**Fig. 5:**
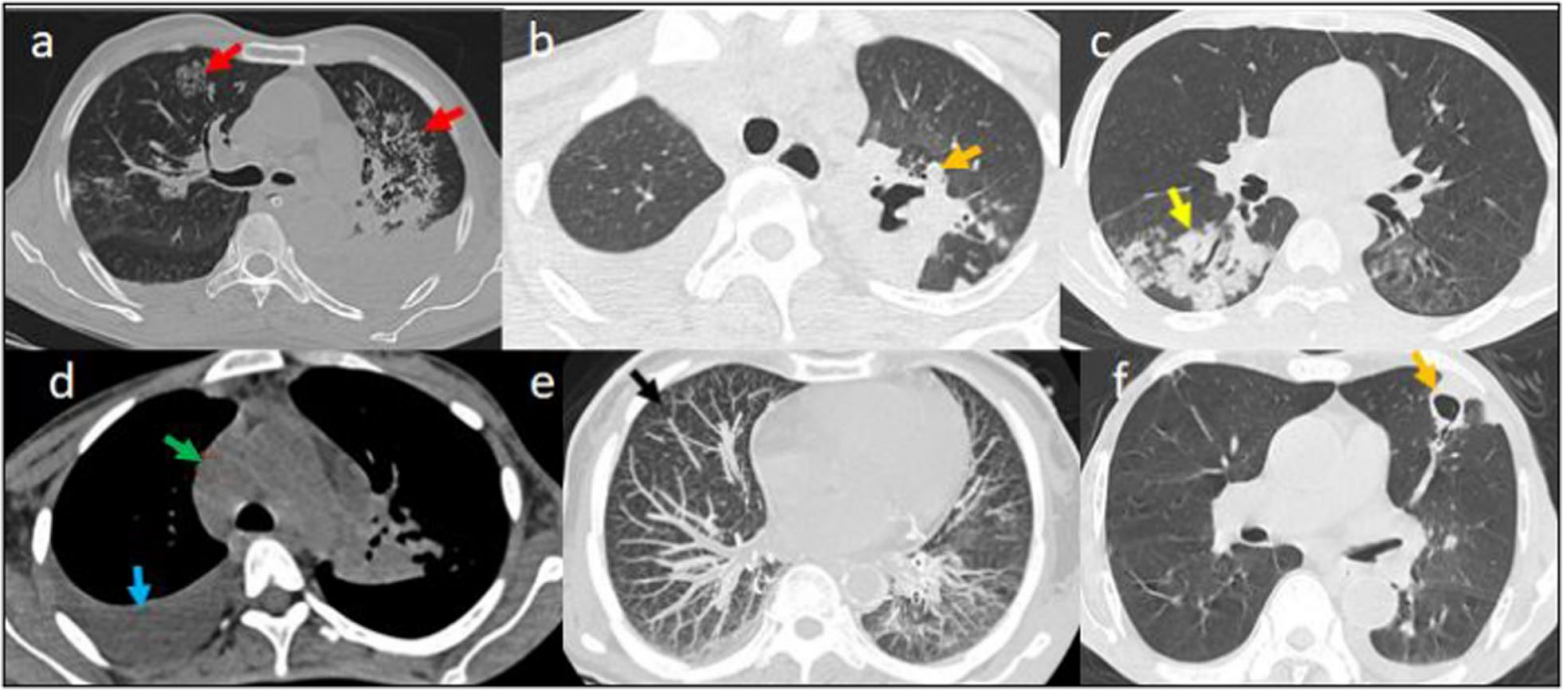
HRCT chest axial sections of different patients showing imaging features of COVID patients mimicking tuberculosis—**a** showing multiple tree in bud nodules (red arrow) in bilateral upper lobes (left > right). **b** Cavity with surrounding consolidation (orange arrow) in apicoposterior segment of left upper lobe. **c** Patchy dense consolidation (yellow arrow) in superior segment of right lower lobe. **d** Enlarged right paratracheal node with right pleural effusion. **e** MIP image showing multiple tiny centrilobular nodules (black arrow). **f** Small cavity with surrounding consolidation (orange arrow) in lingular segment of left upper lobe and showing communication with segmental bronchus

The presence of pulmonary nodules in images of some COVID patients led to the suspicion of associated tuberculosis, which is an endemic in this part of the subcontinent. Atypical category of COVID 19 couldn’t be differentiated from tuberculosis in HRCT chest because of similar imaging features (Fig. 5). Accurate diagnostic tests are essential for both TB and COVID-19. Since sputum could not be collected due to the constraints of fear psychosis and patients’ condition, testing with IGRA for exclusion of TB was resorted to. We conducted a study in 50 COVID patients over a period of 6 months suspicious of tuberculosis. Interferon-gamma release assay (IGRA) test was undertaken. IGRA test was found to be negative in 42 patients and positive in 8 patients. We concluded that it is not always possible to differentiate features of atypical COVID-19 from tuberculosis on the basis of HRCT chest alone. Hence, exclusion of tuberculosis will need supportive relevant laboratory investigations (sputum AFB, CB NAAT and IGRA) for appropriate diagnosis and management.

### Artificial intelligence analysis

**Fig. 6:**
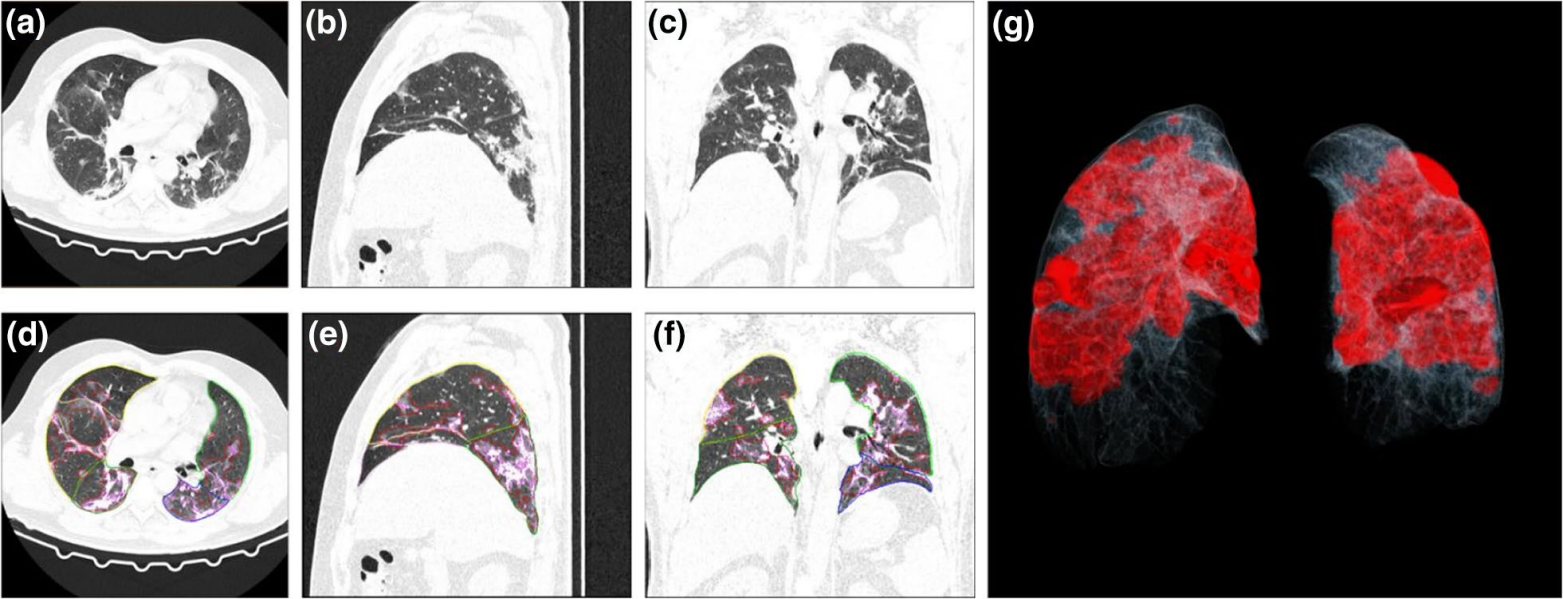
Anonymized HRCT images in axial (**a**), sagittal (**b**) and coronal (**c**) planes. Automated segmentation of lungs, lobes and abnormalities in same slices (**d**, **e**, **f**). 3D representation of lungs with abnormalities (**g**)

**Fig. 7:**
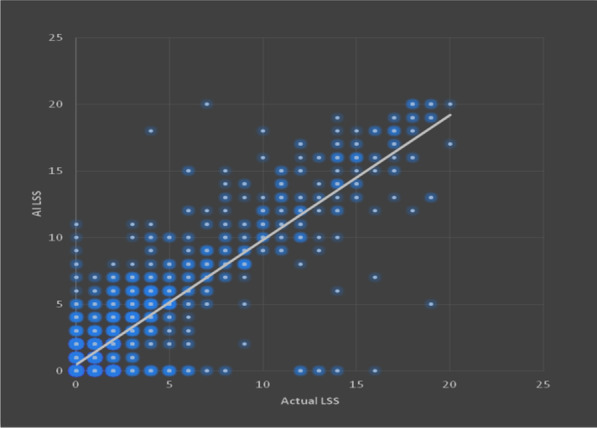
Scatter plot showing linear correlation between AI LSS and manual LSS

**Fig. 8:**
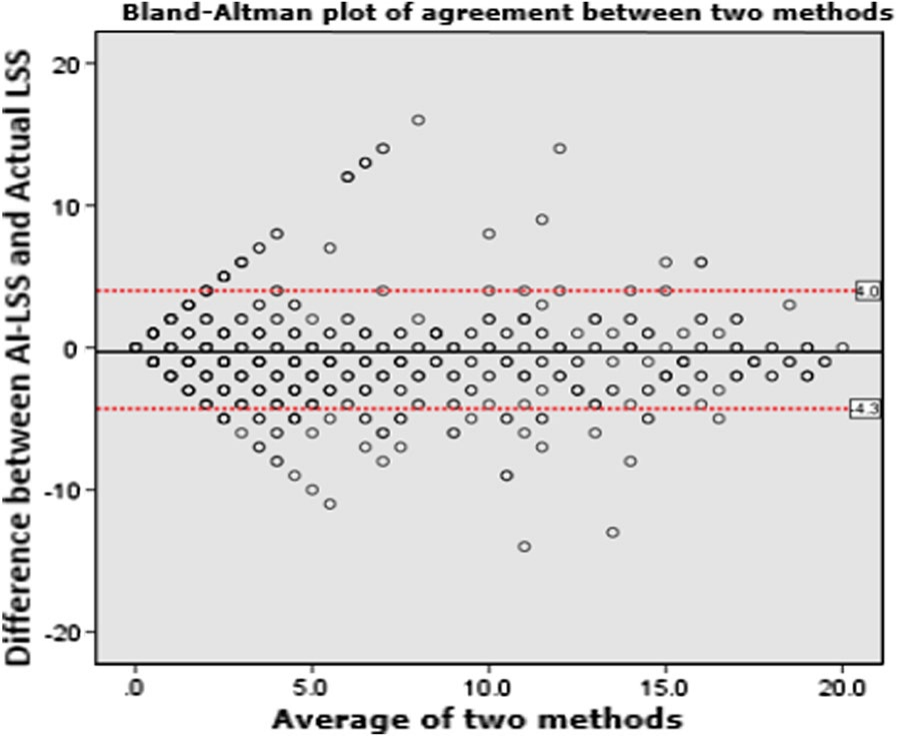
Bland–Altman plot showing good agreement between two methods

Percentage of opacity (PO) and percentage of high opacity (PHO) in HRCT chest were obtained using “*CT pneumonia analysis* algorithm” (Fig. 6). The linear correlation by calculating Pearson’s correlation coefficient (*r*) value was 0.88 which suggests strong linear correlation between the LSS of AI with actual values (Fig. 7). Intraclass correlation coefficient (ICC) and their 95% confidence intervals were calculated to be 0.87, 0.86 and 0.88, respectively. The ICC of 0.8 shows good reliability between actual LSS and LSS assessed by AI-based algorithm. The confidence interval shows 95% of the times ICC will lie between 0.86 and 0.88. Bland–Altman plot shows good agreement between two methods (Fig. 8).

### CT staging with duration of hospital stay and mortality

In our study, 340 (7.8%) patients died during an average follow-up of 14.2 ± 4 days (range 1–24 days). Mortality was higher in patients ≥ 60 years of age (53.3%) than in patients < 60 years of age (46.7%) (*p* < 0.003), with a mean mortality age of 51 years. The average duration of stay in hospital was found to be 12 days (range 9 to 24 days). Patients with CTSS of < 5 had less mean duration of stay (10 days) and did not require any active management. Patients with CTSS of 5 to 15 had mean duration of 13 days; most of them recovered after proper management. Patients with CTSS > 15 were severely ill patients with relatively higher mortality of 32.9%. These patients were kept in ICU.

Correlation of CTSS with laboratory markers was undertaken (Table 5). *p* Value was calculated, and Pearsons’ correlation method was used to find the correlation coefficient (r).

**Table 5:** Correlation of CTSS with laboratory markers

CTSS Correlation with	Pearsons correlation coefficient (*r*)	*p* Value	Correlation
1. D-Dimer (*n* = 1448)	0.567	< 0.001	Moderate significant positive correlation
2. CRP (*n* = 1858)	0.651	< 0.0001	Moderate significant positive correlation
3. ESR (*n* = 1680)	0.290	< 0.001	Mild to moderate significant positive correlation
4. Lymphocyte count (*n* = 2400)	− 0.198	< 0.001	Mild significant negative correlation
5. Procalcitonin (*n* = 1520)	0.188	< 0.001	Mild significant positive correlation
6. WBC count (*n* = 2550)	0.093	> 0.05	Insignificant correlation

**Fig. 9:**
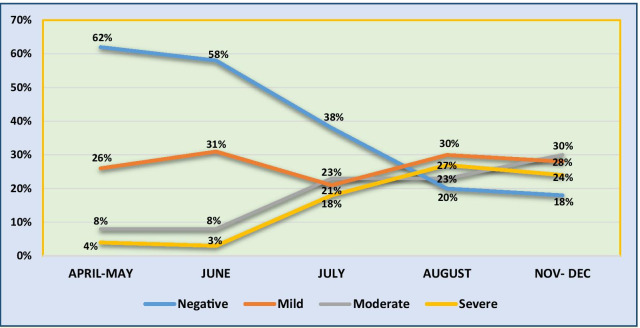
Trend of severity in eastern Indian population. *X*-axis—period in months. *Y*-axis—patient population. Mild, moderate and severe categories were divided on the basis of CT severity score as 1–5, 6–15 and 16–25, respectively

The trend of severity (on the basis of CTSS) in eastern Indian population over the periods of month (from March to December) is shown in (Fig. 9).

### USG thorax in COVID

**Fig. 10:**
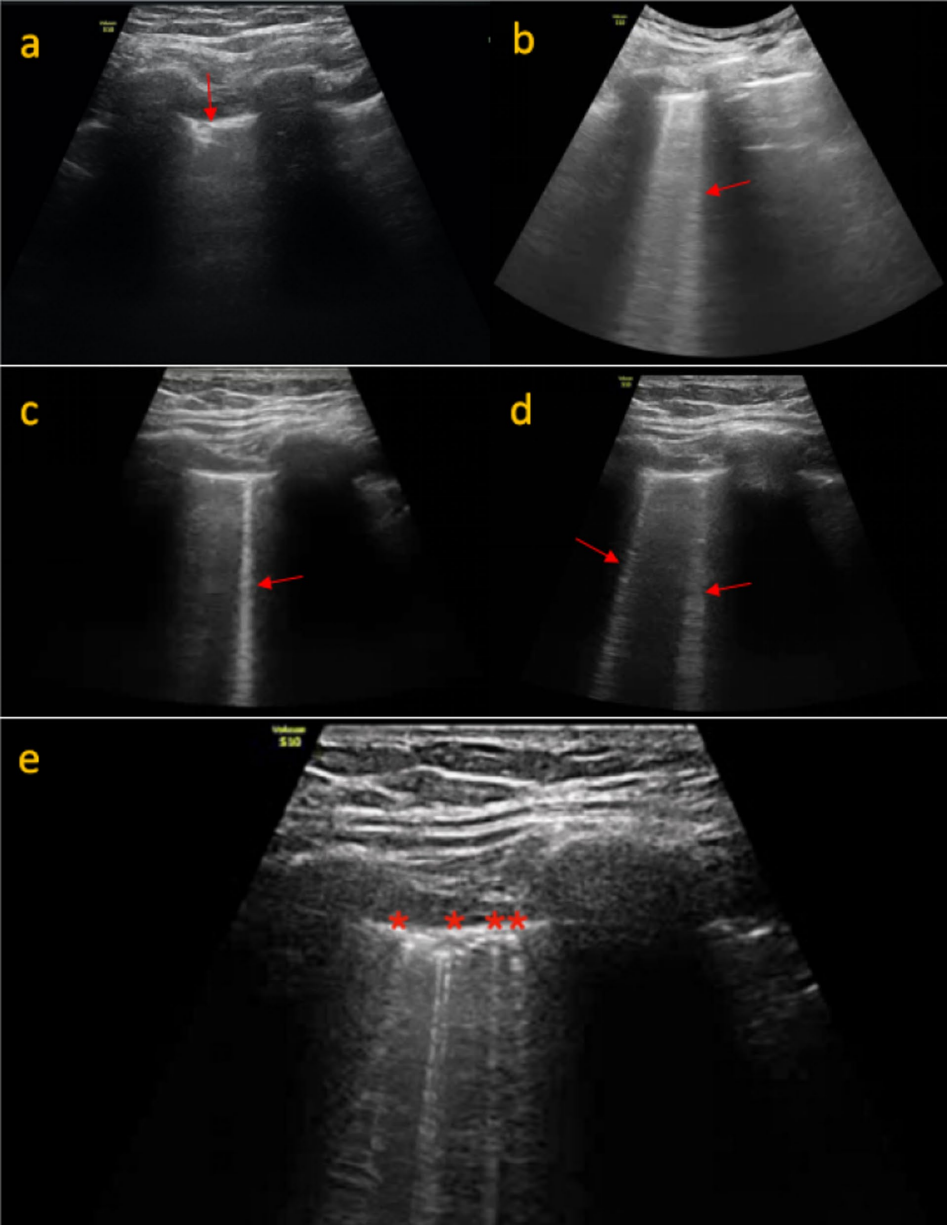
LUS imaging features in COVID-19. **a** Small subpleural consolidation (red arrow) with pleural thickening depicted by a linear high frequency (15 MHz) of the lateral inferior area of the left lung. **b** Classic waterfall sign (red arrow) next intercostal space showing normal A and B lines. **c** Torchlight sign (red arrow) by B lines in the posterior superior area of the right lung scanned by a linear high frequency (15 MHz) transducer. **d** Multifocal B lines (red arrow) coming off from pleura using a curvilinear (3 MHz) transducer in the lateral inferior area of the right lung. **e** Multifocal B lines or separated B line with pleural irregularity (asterisk) scanned by a linear high-frequency (15 MHz) transducer in the anterior superior area of the right lung

We studied LUS in 124 ICU patients. The presence of B lines was the most common finding in 118 (95.16%) of the cases. Pleural line thickening in > 6 lung areas was seen in 98/124 (79.03%). The right lung was affected in 100/124 (80.5%) and the left lung in 96/124 (78.7%). LUS imaging features are shown in Fig. 10.

Confluent B lines originating from regular pleural lines which were previously described as “waterfall” artefacts (Fig. 10b) and were supposed to represent an early stage of actively spreading COVID-19 pneumonia alternating with areas of normal lung parenchyma were observed in 76/124 (61.2%) of the cases.

An interesting finding also encountered in this study was the narrow and bright B line originating from the thickened pleura which was brighter and narrower than the regular separate B lines and did not tend to fan out till the end of the screen. We are uncertain if this is a variety of the already described “waterfall” and “light beam sign” which originate from a larger portion of the regular pleural line. We felt this was similar to a “torchlight” a “single light beam” being flashed in a sense that it was extremely bright undoubtedly brighter than the Z line and B line, was erased by the A lines and almost always was from a thickened pleura. These “torchlight” B lines (Fig. 10c) were observed in 32 (25.8%) of the patients and were seen more in the antero-superior and antero-inferior areas of bilateral lungs typically in clinically severe cases. Further validation will be needed to establish if it can be an indicator of severe COVID pneumonia.

In contrast, separate B lines (> 3 in a single intercostal space) coming off from an irregular pleural lines were evident in most cases 68/124 (54.8%) of the cases. Variable consolidations 32/124 (25.8%) are predominantly seen in the posterior lung areas. Both subpleural and “starry sky” pattern of consolidation (bright infiltrates) was encountered. Pleural and pericardial effusions were seen in 8/124 (6.4%) and 2/124(1.6%), respectively.

### Chest X-ray efficacy and comparison with CT

Total 784/1400 (56%) patients were found to be negative for radiological thoracic involvement. Out of 1400 COVID-positive patients, 294 (21%) showed lung consolidations, 230 (16.5%) presented with GGO, 80 (5.7%) with nodules and 84 (6%) with reticular–nodular opacities. Sensitivity of chest X-ray for different lung abnormalities in COVID-positive patients taking CT chest as a comparing imaging tool (*n* = 1400) is endorsed (Table 6).

**Table 6:** Sensitivity of chest X-ray versus HRCT in different lung abnormalities (*n* = 1400)

Characteristics	No. of findings correctly matched with HRCT	No. of findings not correctly matched with HRCT	Sensitivity (%)
Consolidation	294	126	70
Ground-glass opacities	230	162	59
Pulmonary nodules	80	108	42.5
Pleural effusion	40	26	60.6
Reticular opacities	84	108	43.7
Cavitatory lesion	20	22	47.6
Cardiomegaly	32	10	76.1
Overall sensitivity of CXR in comparison to HRCT			55.1

### SpO2 correlation with CTSS and prediction of hypoxia

A study of 1000 patients was conducted where we tried to find out if SpO2 values correlates with (CTSS) (Table 7)**.** Mean CTSS in this study population was 8.58 ± 6.5. Mean SpO2 values in CTSS Category 1, 2 and 3 were 95.62 ± 3.56; 92.01 ± 4.62; and 87.83 ± 4.92, respectively. We tried to correlate the CTSS of patients with their SpO2 levels at the time of initial scan. Statistical correlation between CTSS and SpO2 levels at the time of initial scan was significant (Pearson’s correlation coefficient (*r*) = − 0.261 and *p* value < 0.01). Negative Pearson’s correlation coefficient suggests the fall in patients SpO2 levels with an increase in CTSS.

**Table 7:** Comparison of CTSS with clinical severity and SpO2 levels at the time of scan and on follow-up

CTSI categories	No. of patients (out of 1000)	Clinical grade	Spo_2_ grading	No. of patients	%	Patients who developed hypoxemia on follow up	Percentage of patients who developed hypoxemia on follow up
0–7	512	Asymp &Mild	> 95	371	72.46	42	11.32
		Moderate	90–94	112	21.87	–*	–
		Severe	< 90	29	5.66	–*	–
8–15	295	Asymp &Mild	> 95	63	21.35	10	15.87
		Moderate	90–94	189	64.06	–*	–
		Severe	< 90	43	14.57	–*	–
16–25	193	Asymp &Mild	> 95	14	7.21	2	14.28
		Moderate	90–94	46	23.71	–*	–
		Severe	< 90	133	69	–*	–

^*^–Not included in follow-up study as these patients were already hypoxemic at the time of admission

Total 448 (44.8%) out of 1000 patients included in this study were having normal SpO2 values at the time of HRCT thorax and rest patients were already hypoxemic (i.e. SpO2 level < 95%). Out of these 448 patients, 371 (82.81%) patients were in CTSS Category 1 and about 63 (14.06%), 14 (3.21%) patients were in CTSS Category 2 and 3, respectively. A number of patients who developed hypoxemia (SpO2 < 95%) on follow-up SpO2 measurements in CTSS Category 1, 2 and 3 were 42 (11.32%), 10 (15.87%) and 2 (14.28%), respectively, i.e. only 54 patients out of 448 patients with normal SpO2 levels developed hypoxemia on follow-up. The association between CTSS and development of hypoxemia based on follow up SpO2 levels was statistically found to be improbable (Chi-square value = 1.21, degree of freedom (*df*) 2 and *p* value = 0.570).

## Discussion

HRCT chest findings were analysed and correlated with symptomatology and severity of patients to explore possible roles of HRCT in diagnostic, therapeutic and prognosis of COVID-19 disease. A total of 4338 images were studied. Out of asymptomatic patients, 39.8% were found to be radiologically positive. This significant percentage of asymptomatic COVID-19 patients pose a real threat to society as they may act as silent carriers for transmission of virus.

The prominent HRCT chest features were bilateral ground-glass opacities (GGOs) with or without consolidation. In this study, nearly two-third patients had positive CT findings with most patients (55.8%) showing a mixed pattern of GGO and consolidation. The distribution of the lesions in our study was mostly peripheral and lower lobar with multilobar and bilateral lung involvement seen in 66% patients. The predilection for these areas has also been reported previously [16]. Thin or thick subpleural band-like opacities usually in the basal segments of lower lobes and some specific patterns like interstitial/septal thickening (crazy paving/reticular pattern), reverse halo sign and halo sign were seen in a significant proportion of patients. These findings corroborate with the findings of the study done by Ye et al. [17].

Positive HRCT chest findings were also seen in some asymptomatic patients, especially the young, may be due to the differences in disease tolerability and immune status. 91.3% of cases having score ≤ 7 were asymptomatic or mild. On the other hand, 75.1% cases having CT severity score 8–14 were clinically moderate and 81.2% cases having score ≥ 15 were clinically severe. The CT severity scores (CTSS) showed good correlation with clinical status of patients, as seen in previous studies [18]. It was also observed that clinically severe patients showed higher CTSS compared to the non-severe patients. The positive predictive values (PPVs) of CTSS ≥ 15 for detecting clinically severe and symptomatic patients are 81.2% and 98.5%, respectively.

These data suggested that CTSS may be an informative indicator to predict the severity of the disease. The proposed grades of CTSS are (1) mild (CTSS: 0–7), (2) moderate (CTSS: 8–14) and (3) severe (CTSS: 15–25). This grading can help to prognosticate and tailor the clinical management of patients. HRCT chest scans of patients in the early phase (0–7 days) of disease show mostly GGO and in late stage (> 14 days) consolidation tends to be a predominant feature. In the early stages, single or multiple small GGO, consolidation and mild interstitial thickening could be seen. In the intermediate phase (8–14 days), the number of involved lobes was more with increase in consolidation and septal thickening in the involved lobes which correlated with increased disease severity. The diffuse lesions involving both peripheral and central parts of lungs, causing “white lungs,” appearance were seen in the most severely affected patients.

The other observation in our study was that, even if the total area of lung involved remained the same, features like increased reticulation, predominance of consolidation, presence of fibrous stripes and development of bronchiectatic changes indicated late stage. This corroborated with clinical status. A long-term follow-up is needed to detect complete resolution or otherwise. The data of our study indicate that CT findings vary according to the time of scan, from the onset of illness. This reiterates the results observed by Bernheim et al. who suggested progression of disease in the form of GGOs in early stage to crazy paving/reticulation and consolidation in later stages [19]. Computed tomography chest imaging also showed some non-specific findings which include pleural effusion, pulmonary nodules and thoracic lymphadenopathy. Majority of cases with pleural effusion were in the intermediate phase of the disease.

On LUS, confluent B lines originating from regular pleural lines which were supposed to represent an early stage of actively spreading COVID-19 pneumonia were observed in 76/124 (61.2%) of the cases; however, other studies which were not in the ICU setting reported a higher incidence of confluent B lines [13, 14, 20]. In our study, the subpleural kind of consolidation was more common than the “starry sky” pattern. This was a deviation from the previous studies which reported a higher incidence of the “starry sky” pattern usually involving more of the lung parenchyma [20, 21].

In our study, 976 patients had pre-existing comorbidities like diabetes mellitus (DM) (*n* = 640), hypertension (HTN) (*n* = 342), pulmonary tuberculosis (PTB) (*n* = 107), asthma (*n* = 98), chronic kidney disease (CKD) (*n* = 60), cardiac disease (*n* = 62), others including stroke and hypothyroidism (*n* = 60). Among these, DM was the most common followed by HTN [22, 23]. The impaired immunity, hypertension and chronic kidney disease, due to hyperglycaemia and the chronic inflammatory state in case of diabetes, lead to increased risk of development of cytokine storm and severe disease [24–27].

It was reported that approximately 50 percent of patients were confirmed to have elevated D-dimer levels and that high D-dimer rates correlated with poor prognosis [28, 29]. Moderate positive correlation (*r* = 0.56) with a *p* value of < 0.001 remained as supporting biomarker in our series. Pearson’s correlation of C-reactive protein (CRP) levels in 1858 patients indicated *p* value of < 0.0001, i.e. moderately positive and useful predictor of illness. Levels of CRP were found to be increased in 74% cases with mean CRP levels of 78 mg/L (Range 24 -223 mg/L) among the total deaths. Procalcitonin is now being seen to be an important indicator for early detection low risk of bacterial co-infection in the ongoing COVID-19 pandemic [28]. Our observation also suggests mild positive correlation of procalcitonin and ESR in determining the severity of COVID, but since the correlation is mild, both cannot be considered as reliable markers of the severity. Considerable proportion of patients showed lymphopenia. Associated and iatrogenic complications of the disease like pneumomediastinum and pneumothorax were well picked up on HRCT for early management.

Manual assessment of chest CT in COVID-19 pandemic scenario was relatively more stressful and time-consuming. Hence, there was a need for rapid, precise and automated method of detection and quantification of common findings of COVID-19 in chest CT. The “CT Pneumonia Analysis” AI algorithm gives multiple quantitative parameters which are helpful in assessing the extent of individual lobe involvement as well as combined whole lung involvement. The Pearson’s correlation coefficient showed strong linear relationship between both methods in assigning LSS. Intraclass correlation coefficient showed good reliability and Bland–Altman plot showed good agreement between AI LSS and actual LSS reported manually. However, the software (“CT Pneumonia Analysis”) could not detect GGOs in early stage of disease and also other *atypical* findings like pleural and pericardial effusions, mediastinal lymphadenopathy, nodules and complications like pneumothorax, pneumomediastinum, surgical emphysema in contrary to conventional method. It needs appropriate modifications and training for detecting these additional features.

There was a trend of increasing severity from month of July onwards as compared to months of April to June. In April–May the proportion of patient having CTSS 16–25 was 4%, which touched 27% in August and September. It slightly decreased to 24% during last quarter. The sudden change in trend of severity could be attributed to the migrants labourers, domestic and international travellers, who came back post lockdown. Our assumption was that this category of patients could have been infected by more virulent viral strain.

### Limitations

1. We examined all the main radiological modalities, with the exception of lung MRI, which has a debatable usefulness, especially in COVID-19.
2. The AI software which we used in our study was unable to recognize atypical features of COVID-19.

## Conclusions

COVID-19 could be well classified as typical, atypical and indeterminate categories. Diabetics, aged population, lower socioeconomic strata, males were more vulnerable.

HRCT thorax is now an established technique for instant diagnosis of COVID-19. It also helps in faster triage and identifying the patients for ICU care, thus reducing the wastage of skilled manpower, hospital beds and other precious resources. RT-PCR and laboratory support are important aspects of patient management. Bed-side X-rays and ultrasonography played a limited role in the ICU setting, where a chunk of the patients could not be shifted for CT scan.

The possibility of predicting impending or progression of hypoxia was not possible when SpO2 mapping was correlated with the CT severity score. AI was alternatively tried with available software (*CT pneumonia analysis*) which was not so appropriate considering the imaging patterns in the bulk of *atypical category*. Hence, it is pertinent to use an appropriate software for faster analysis of the images targeting higher accuracy.

### Recommendations

1. HRCT thorax should be undertaken on the first visit/hospitalization if COVID positive. Repeat after 7–10 days depending on the outcome/status of recovery, then follow-up in 3 months and 6 months to assess the residual damage.
2. Availability of exclusive CT scanner for COVID hospital and PACS will help in non-interference with the non-COVID population in a busy tertiary care hospital, especially in this kind of pandemic situation. Instant diagnostic capability with HRCT for early triage is a boon.
3. Thorough knowledge of imaging features and reporting criteria/protocol will add to the accuracy of evaluation and management.
4. Use of AI with appropriate software can speed up the reporting and analysis during the pandemic situation.
5. Radiography and USG in non-ambulatory patients are good options.

## Data Availability

The datasets generated and/or analysed during the current study are not publicly available due [COVID data are monitored by the government] but are available from the corresponding author on reasonable request.

## Abbreviations

HU: Hounsfield unit
AI: Artificial intelligence
LUS: Lung ultrasound
rRT-PCR: Real-time reverse transcription polymerase chain reaction
HRCT: High-resolution computed tomography
CTSS: CT severity score
MPR: Multiplanar reformatting
DI2IN: Deep image-to-image network
LSS: Lung severity score
LHOS: Lung high opacity score
PO: Percentage of opacity
PHO: Percentage of high opacity
ICC: Interclass correlation
LUSS: Lung segmental scores
GGO: Ground-glass opacity

## References

[1] Li Y, Xia L (2020). Coronavirus disease 2019 (COVID-19): role of chest CT in diagnosis and management. Am J Roentgenol.

[2] Zhu H, Wei L, Niu P (2020). The novel coronavirus outbreak in Wuhan, China. Glob Health Res Policy.

[3] Rubin GD, Ryerson CJ, Haramati LB, Sverzellati N, Kanne JP, Raoof S (2020). The role of chest imaging in patient management during the COVID-19 pandemic: a multinational consensus statement from the Fleischner Society. Radiology.

[4] Kovács A, Palásti P, Veréb D, Bozsik B, Palkó A, Kincses ZT (2021). The sensitivity and specificity of chest CT in the diagnosis of COVID-19. Eur Radiol.

[5] Shi F, Wang J, Shi J, Wu Z, Wang Q, Tang Z, Shen D (2020). Review of artificial intelligence techniques in imaging data acquisition, segmentation and diagnosis for covid-19. IEEE Rev Biomed Eng.

[6] Xu YH, Dong JH, An WM (2020). Clinical and computed tomographic imaging features of novel coronavirus pneumonia caused by SARS-CoV-2. J Infect.

[7] Xie X, Zhong Z, Zhao W, Zheng C, Wang F, Liu J (2020). Chest CT for typical 2019-nCoV pneumonia: relationship to negative RT-PCR testing. Radiology.

[8] Simpson S, Kay FU, Abbara S, Bhalla S, Chung JH, Chung M, Henry TS, Kanne JP, Kligerman S, Ko JP, Litt H (2020). Radiological Society of North America Expert Consensus Statement on Reporting Chest CT Findings Related to COVID-19. Endorsed by the Society of Thoracic Radiology, the American College of Radiology, and RSNA - Secondary Publication. J Thorac Imaging.

[9] Ghesu FC, Georgescu B, Zheng Y, Grbic S, Maier A, Hornegger J, Comaniciu D (2017). Multi-scale deep reinforcement learning for real-time 3D-landmark detection in CT scans. IEEE Trans Pattern Anal Mach Intell.

[10] Yang D, Xu D, Zhou SK, Georgescu B, Chen M, Grbic S, Metaxas D, Comaniciu D (2017) Automatic liver segmentation using an adversarial image-to-image network. In: International conference on medical image computing and computer-assisted intervention. Springer, Cham, pp 507–515

[11] Chaganti S, Balachandran A, Chabin G, Cohen S, Flohr T, Georgescu B, Grenier P, Grbic S, Liu S, Mellot F, Murray N (2020) Quantification of tomographic patterns associated with COVID-19 from chest CT. arXiv preprint arXiv:2004.01279 10.1148/ryai.2020200048 PMC7392373 33928255

[12] World Health Organization (2020) Clinical management of severe acute respiratory infection when novel coronavirus (nCoV) infection is suspected—interim guidance. WHO, Geneva. https://www.who.int/publications-detail/clinicalmanagement-of-severe-acute-respiratory-infection-when-novelcoronavirus-(ncov)-infection-is-suspected.

[13] Volpicelli G, Gargani L (2020). Sonographic signs and patterns of COVID-19 pneumonia. Version 2. Ultrasound J.

[14] Volpicelli G, Lamorte A, Villén T (2020). What’s new in lung ultrasound during the COVID-19 pandemic. Intensive Care Med.

[15] Manivel V, Lesnewski A, Shamim S, Carbonatto G, Govindan T (2020). CLUE: COVID-19 lung ultrasound in emergency department. Emerg Med Australas.

[16] Lomoro P, Verde F, Zerboni F, Simonetti I, Borghi C, Fachinetti C (2020). COVID-19 pneumonia manifestations at the admission on chest ultrasound, radiographs, and CT: single-center study and comprehensive radiologic literature review. Eur J Radiol Open.

[17] Ye Z, Zhang Y, Wang Y, Huang Z, Song B (2020). Chest CT manifestations of new coronavirus disease 2019 (COVID-19): a pictorial review. Eur Radiol.

[18] Zhang J, Meng G, Li W, Shi B, Dong H, Su Z (2020). Relationship of chest CT score with clinical characteristics of 108 patients hospitalized with COVID-19 in Wuhan, China. Respir Res.

[19] Bernheim A, Mei X, Huang M, Yang Y, Fayad ZA, Zhang N (2020). Chest CT findings in coronavirus disease-19 (COVID-19): relationship to duration of infection. Radiology.

[20] Alharthy A, Faqihi F, Abuhamdah M, Noor A, Naseem N, Balhamar A, Al Saud AA, Brindley PG, Memish ZA, Karakitsos D, Blaivas M (2020). Prospective longitudinal evaluation of point-of-care lung ultrasound in critically Ill patients with severe COVID-19 pneumonia. J Ultrasound Med.

[21] Ai T, Yang Z, Hou H (2020). Correlation of chest CT and RTPCR testing in coronavirus disease 2019 (COVID-19-19) in China: a report of 1014 cases. Radiology.

[22] Emami A, Javanmardi F, Pirbonyeh N, Akbari A (2020). Prevalence of underlying diseases in hospitalized patients with COVID-19: a systematic review and meta-analysis. Arch Acad Emerg Med..

[23] Yang J, Zheng Y, Gou X, Pu K, Chen Z, Guo Q (2020). Prevalence of comorbidities in the novel Wuhan coronavirus (COVID-19) infection: a systematic review and meta-analysis. Int J Infect Dis.

[24] Guo W, Li M, Dong Y, Zhou H, Zhang Z, Tian C (2020). Diabetes is a risk factor for the progression and prognosis of COVID-19. Diabetes Metab Res Rev.

[25] Blazer S, Khankin E, Segev Y, Ofir R, Yalon-Hacohen M, Kra-Oz Z (2002). High glucose-induced replicative senescence: point of no return and effect of telomerase. Biochem Biophys Res Commun.

[26] Zuin M, Rigatelli G, Zuliani G, Rigatelli A, Mazza A, Roncon L (2020). Arterial hypertension and risk of death in patients with COVID-19infection: systematic review and meta-analysis. J Infect.

[27] Lippi G, Wong J, Henry BM (2020). Hypertension in patients with coronavirus disease 2019 (COVID-19): a pooled analysis. Pol Arch Intern Med.

[28] Guan WJ, Ni ZY, Hu Y, Liang WH, Ou CQ, He JX, Liu L, Shan H, Lei CL, Hui DS, Du B (2020). Clinical characteristics of coronavirus disease 2019 in China. N Engl J Med.

[29] Arachchillage DR, Laffan M (2020). Abnormal coagulation parameters are associated with poor prognosis in patients with novel coronavirus pneumonia. J Thromb Haemost.

